# Environmental Dyeing and Functionalization of Silk Fabrics with Natural Dye Extracted from Lac

**DOI:** 10.3390/molecules29102358

**Published:** 2024-05-16

**Authors:** Qinru Huang, Zhao Wang, Liwei Zhao, Xiaojuan Li, Haohao Cai, Shuang Yang, Maoli Yin, Jian Xing

**Affiliations:** School of Textile and Garment, Anhui Polytechnic University, Wuhu 241000, China; 13955332396@139.com (Q.H.); 15855165517@163.com (Z.W.); 19832160647@163.com (L.Z.); lixiaojuan@ahpu.edu.cn (X.L.); haoyi173@foxmail.com (H.C.); ys92@sina.com (S.Y.)

**Keywords:** silk, lac, dyeing, mordant, functionality

## Abstract

Most traditional synthetic dyes and functional reagents used in silk fabrics are not biodegradable and lack green environmental protection. Natural dyes have attracted more and more attention because of their coloring, functionalization effects, and environmental benefits. In this study, natural dyes were extracted from lac and used for coloring and functionalization in silk fabrics without any other harmful dyes. The extraction conditions were studied and analyzed by the univariate method. The optimal extraction process was that the volume ratio of ethanol to water was 60:40 with a solid–liquid ratio of 1:10, and reacting under the neutrality condition for 1 h at 70 °C. Silk fabric can be dyed dark owing to the certain lifting property of lac. After being dyed by Al^3+^ post-medium, the levels of the washing fastness, light fastness, and friction fastness of silk fabric are all above four with excellent fastness. The results show that the dyed silk fabrics have good UV protection, antioxidation, and antibacterial properties. The UV protection coefficient UPF is 42.68, the antioxidant property is 98.57%, and the antibacterial property can reach more than 80%. Therefore, the dyeing and functionalization of silk fabrics by utilizing naturally lac dyes show broad prospects in terms of the application of green sustainable dyeing and functionalization.

## 1. Introduction

In recent years, with the improvement in people’s living standards, the awareness of environmental protection, healthy and ecological habits have gradually become popular, and people pay more and more attention to human health. The potential unsafe factors of many chemical synthetic dyes and textile finishing agents have aroused widespread concern [1,2,3]. In the context of the global advocacy of green sustainable development, more and more researchers are committed to replacing synthetic chemicals with green resources without affecting the quality and efficiency of production. Natural dyes not only have the advantages of environmental protection, safety, good biodegradability, and environmental compatibility, but also have certain functions (such as antibacterial, antioxidant, UV protection, etc.), so the application of natural dyes partially replacing synthetic dyes and synthetic finishing agents has received great attention [4,5,6,7]. Many researchers have studied dyeing and functionalizing textiles using natural materials from various sources to develop high value-added textiles [8,9,10].

Silk fabrics are made by natural fibers that are light, soft, and fine in nature, known for their “human second skin” reputation. Owing to their outstanding thermal insulation and moisturizing properties, natural environmental protection, health and comfort, etc., silk fabrics are widely used in daily clothing and home textile industry, especially in intimate clothing and infant clothing [11,12,13]. Although the industrial application technology of silk fabrics is very mature, some challenges need better solutions. For example, silk fabrics are often colored with chemically synthesized acid dyes, which can reduce the sustainability of silk fabrics. In addition, the functionality of silk fabrics (such as anti-ultraviolet, antibacterial, antioxidant) is insufficient, which limits its application in protective clothing, medical dressings, etc., and some synthetic additives are often used to give them functionality [14]. These chemically synthesized dyes and auxiliaries will not only reduce the sustainable development of silk fabrics, but some reagents will also have certain harmful effects on the environment [15]. Therefore, there is great practical value in using natural dyes with certain functions in the silk fabrics to give them color and function.

Lac, also known as shellac, red gum, purple rivet, is a purple natural resin secreted by the female of lac insect in tree branches, mainly containing lac resin, lac wax, and lac pigment [16,17,18]. Lac as a dyeing material mainly plays a role in the lac pigment. Lac pigment is a mixture of anthraquinone carboxylic acids, containing lac acid A, B, C, D, and E five forms, of which lac acid A accounts for more than 85% [19,20]. Lac pigment shows good antibacterial, antioxidant, antiviral, and anticancer effects due to the fact it contains the phenolic hydroxyl group, carbonyl group, and carboxylic group [21]. Lac pigment, as a natural red pigment, has the characteristics of a bright color and being harmless to the human body. Therefore, it is used for textile dyeing, which can meet the requirements of ecology, environmental protection, and health.

**Figure 1:**
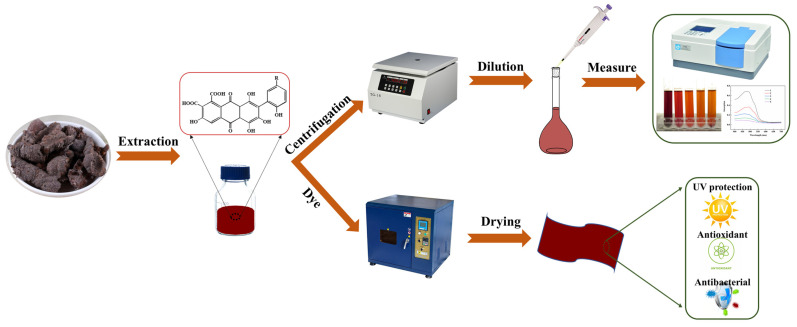
Schematic presentation of lac pigment extraction and dyeing process.

Based on the author’s previous research on traditional natural material dyeing technology in Litang County, Ganzi Prefecture, Sichuan Province, in this study, lac pigment was extracted and used for the functional dyeing of silk (Figure 1). The influence of the solvent system, solid–liquid ratio, pH, temperature, dyeing time, and other factors on the extraction conditions was studied by the univariate method to optimize the extraction process. The dyeing power and mordant properties of lac on silk fabrics were studied, and the fastness (wash resistance, light resistance, friction resistance), UV protection, antioxidation, and antibacterial properties were evaluated to verify its functional properties. This research will open up a new avenue for the dyeing and functionalization of silk fabrics, contributing to a sustainable environment.

## 2. Results and Discussion

### 2.1. Optimization of Extracting Process of Lac Pigment

**Figure 2:**
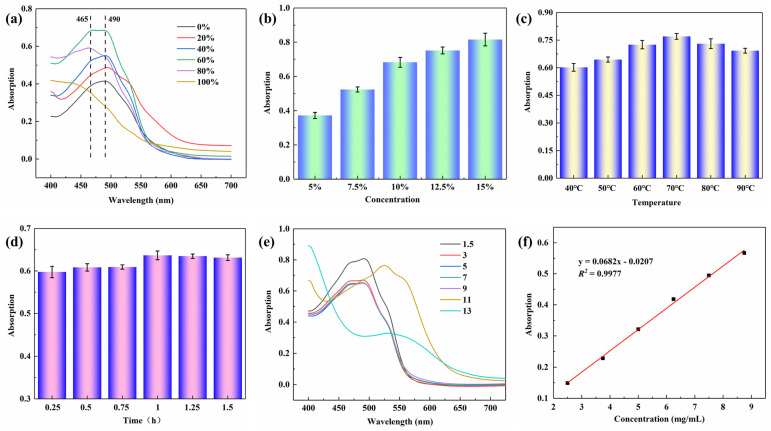
Analysis of the pigment extraction process of lac dye: (**a**) ethanol volume fraction; (**b**) solid–liquid ratio; (**c**) temperature; (**d**) time; (**e**) pH value; and (**f**) absorbance standard curve of the lac dye extract solution.

The nature of the extraction solvent system determines the extraction ability of natural pigments. The use of polar organic solvents can increase the partition coefficient of pigment molecules in the organic extraction solvent to increase the extraction ability of the solvent [3,22]. The absorption peak at 490 nm in Figure 2a is the main characteristic absorption peak of lac pigment [17]. As can be seen from Figure 2a, as the other process conditions were kept stable (solid–liquid ratio 10%, temperature 70 °C, time 1.0 h, pH = 7), the extraction efficiency of the plain color of the lac pigment was significantly improved by the increase in ethanol volume ratio in the solvent system. On the one hand, some of the pigments in the lac pigments (red lac) are soluble in ethanol rather than in water. On the other hand, ethanol has a lower surface tension and may produce higher hollowing, causing the cell to rupture and release more dye molecules into the solvent [3,23]. When the ethanol volume fraction is greater than 60%, the absorbance of the extracted solution no longer increases due to the increase in the ethanol volume ratio. Therefore, in the subsequent single factor analysis, all experiments were carried out using 60% ethanol blend extraction solvent.

Figure 2b shows the effect of the solid–liquid ratio on the absorbance of the pigment extracted from lac (temperature 70 °C, time 1.0 h, pH = 7). The results show that the absorbance of the solution increases with the increase in solid–liquid ratio. However, the increase in absorbance decreases as long as the solid–liquid ratio is greater than 10%.

Figure 2c displays the effect of different extraction temperatures on the absorbance of pigments from lac (solid–liquid ratio 10%, time 1.0 h, pH = 7). As the temperature increases, the absorbance of the extract increases, and higher temperatures can increase the kinetic energy of the molecules, allowing more pigments to enter the solvent. When the temperature is higher than 70 °C and continues to rise, part of the resin in the velvet will dissolve, resulting in an increase in solution viscosity, which is not conducive to the extraction of pigment [23].

Figure 2d shows that, when other parameters remain unchanged (solid–liquid ratio 10%, temperature 70 °C, pH = 7), the absorbance of the extraction solution increases with the increase in extraction time. After a certain time, the absorbance of the extraction solution basically remains unchanged or slightly increases, and basically reaches saturation.

Figure 2e exhibits the effect of pH of the solvent system on the absorbance of the pigment extracted from lac (solid–liquid ratio is 10%, temperature is 70 °C, and time is 1.0 h). When the pH value is 1.5, the absorbance of the extracted liquid reaches the maximum in function of the extraction amount. The absorbance of the extract decreases slightly when the pH is in the range of 3–7. This is mainly because it is extracted from the lac pigment, which is a mixture composed of a variety of anthraquinone carboxylic acid compounds. Under alkaline pH conditions, the maximum absorption peak of the pigment of comfrey antler is significantly shifted. The main reason is that the plain color of the comfrey antler is unstable under alkaline conditions, and molecules are easy to rearrange, resulting in color change and maximum absorption peak shift [18,21].

The experimental analysis of the above single factor optimization process parameters showed that, under the solvent system of 60% ethanol solution, considering the time, energy consumption, cost, and complexity of operation, the remaining factors were set at the solid–liquid ratio of 10%, temperature of 70 °C, time of 1.0 h, and pH of 7, respectively. In addition, the standard curve of absorbance and dye concentration at λ_max_ was drawn based on the maximum absorbance in the range of the visible spectrum in the lac pigment extract at 490 nm (Figure 2f), which laid a foundation for the subsequent study on the dyeing process of lac pigment solution on fabrics.

**Figure 3:**
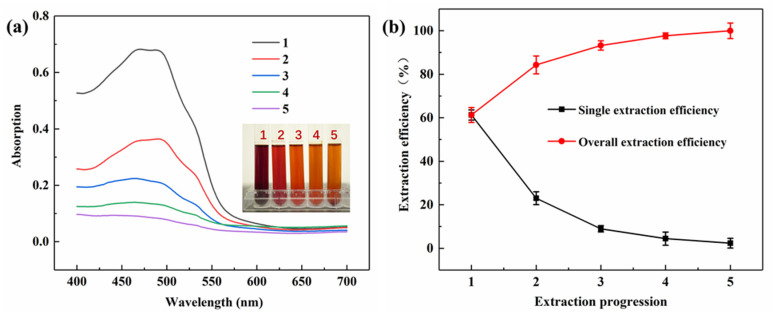
(**a**) Effect of lac extraction order on absorbance; (**b**) Single extraction efficiency and overall extraction efficiency of different extraction stages.

Considering the fact that there were many pigments left in the residue of the comfrey antler after one extraction, the extraction rate of the lac pigment was analyzed by the extraction order experiment. The effects of the extraction stages on the absorbance of the extraction solution are shown in Figure 3a, whilst the effects of the extraction rate and the total extraction rate of the corresponding extraction stages are shown in Figure 3b. Nearly 93.23% of the pigment in the lac has been dissolved in the solvent system after three extraction times, and the amount of pigment extracted shows no increase as the extraction stages continue to increase. Considering the production cost and resource utilization, the extraction stages of comfrey antler pigment should be controlled at three times.

### 2.2. Dyeing Fabrics by Lac Extracts

**Figure 4:**
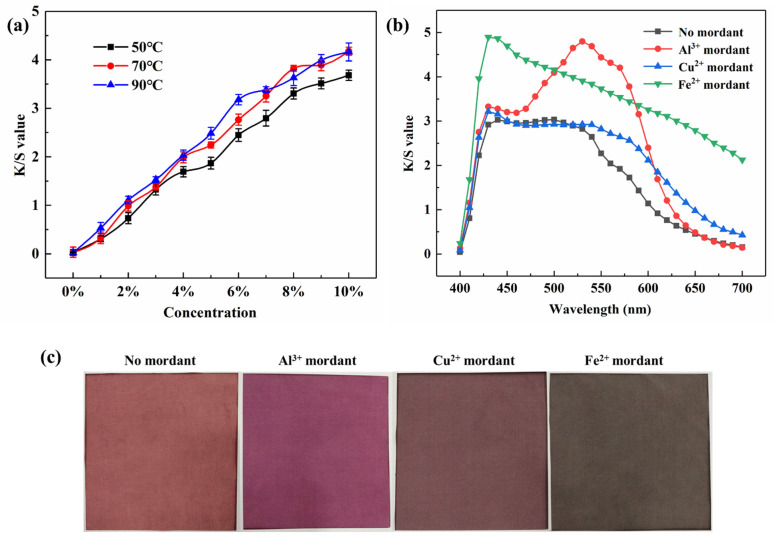
Effect of (**a**) dye concentration and (**b**) mordant on the K/S value of silk fabrics; (**c**) Representative images of silk fabrics.

The lifting properties of natural dyes are very important for dark dyeing and practical applications, and the dyes with good enhancing properties can dye stronger colors on textiles. Considering the molecular structure characteristics of the lac pigment, two process parameters (concentration and temperature) were selected to study the lifting ability of lac pigment on dyeing the silk fabrics. It can be seen from Figure 4a that the K/S value of the fabrics increase with the increase in the mass fraction of the lac extract (in the range of 0~10%), and the color of the fabrics is gradually improved. In this concentration range, the lac extract shows a certain lifting force on the dyeing of silk fabrics, and is suitable for a darker color.

In addition, with the increase in temperature, the K/S value of silk fabrics increased. The dye concentration is large (7–10%), the K/S value remains unchanged with the dyeing temperature rising from 70 °C to 90 °C. Due to the low temperature, the lac pigment molecules show higher aggregation degree, the swelling degree of silk fibers is lower, and the diffusion rate is lower during the lac pigment molecules in silk fibers. With the increase in temperature, the swelling degree of silk fibers increased; meanwhile, the thermal movement of the lac pigment molecules increased, the diffusion rate of the lac pigment in silk fibers increased, the dyeing rate increased, and the K/S increased [24,25]. When rising to a certain temperature, the dyeing reached equilibrium; despite increasing the temperature, the adsorption amount of pigment balance remained stable, and the K/S was unchanged.

The lac dyed silk fabrics were post-mordant treated with aluminum, copper, and ferrous metal ions, and the results of color changes were characterized by absorption spectrum curves, as well as L*, a*, and b* values, as shown in Figure 4b and Table 1. After three metal ions mordant treatment, the color depth value decreases and the color deepens (Figure 4c). Unmortified shellac dyed fabrics have a maximum absorption wavelength at 440 nm. After aluminum ion treatment, the wavelength of the maximum absorption peak moves to the longer wave, which means that the color moves to the red direction a. The b* value of aluminum ion mordant fabrics change to negative, indicating that its blue light component is heavier [26]. After copper treatment, the maximum absorption wavelength nearly unchanged, and only L*, a*, and b* decreased, whilst the color brightness of fabrics also decreased. After ferrous ion treatment, the dyed fabrics showed extensive absorption in the visible region, indicating that the mordant fabrics exhibited a darker color [27]. The color coordinate (a*, b*) of the ferrous ion mordant fabrics is closer to the origin, which also proves that the color of mordant fabrics is darker (Figure 4c) [28].

### 2.3. Color Fastness of Dyed Fabrics

Silk fabrics, in the process of use, are subjected to a series of mechanical effects, such as friction, washing, light, and so on. In practical applications, silk fibers have different color fastness requirements due to different uses. the fabrics dyed by natural dyes usually lead to a low color fastness problem in dyed textiles, and the mordant dyeing method is used to overcome this problem [29,30,31]. In this study, 8% lac extract was dyed and 2% mordant was used, and the color fastness of the dyed silk fabrics is shown in Table 2. It can be seen from Table 2 that mordant dyeing can significantly improve the color fastness of silk, especially the washing color fastness. The washing color fastness of lac dyed silk fabrics is very poor, only in grade 2. The poor washing color-changing fastness of lac is due to its good water solubility and two containing carboxyl groups. After mordant dyeing, the washing color fastness and friction of the fabrics are improved, benefiting from the complexes between the silk fibers and lac pigment during the process of mordant dyeing, which improves the binding force between the dye and the fibers. In addition, the water solubility of the dye decreases after the coordination reaction [4,10]. The lac dyed silk fabrics show good light fastness, reaching level 4. The main reason for this phenomenon is that the chromogenic group in the lac dye within an anthraquinone structure is difficult to photo-oxidate under ultraviolet and visible light irradiation, exhibiting good light stability and high sunlight fastness [21].

**Figure 5:**
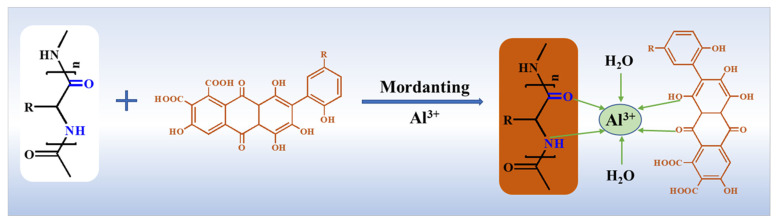
Formation and fixation mechanism of dyeing silk fabrics with lac extract using mordant.

According to GB 18401-2010 “National Basic Safety Technical Code for Textile Products” [32], the color fastness of the lac-dyed silk fabrics after mordant dyeing meets the requirements of washing resistance and friction resistance (minimum level 3). The effects of metal salt mortification on the K/S value, color characteristic value, and color fastness were analyzed comprehensively. The aluminum ion was the best choice for the mortification of lac dyed silk fabric. In the mordant dyeing process, the interaction mechanism between aluminum ion, dye and silk fabrics is shown in Figure 5. The phenolic hydroxyl groups in the molecular structures of lac pigment can form a stable complex with aluminum ion. On the other hand, since the coordination number of the aluminum ion is 6, other coordination sites can form coordination bonds with the amide groups on the silk fabrics, thereby forming coordination complexes between the dye and the silk fabrics [33,34]. 

### 2.4. Functionality of Dyed Fabrics

#### 2.4.1. UV Protection

The effects of different mordants on the UV resistance of silk fabrics are shown in Table 3. The silk fabrics are light and have a poor UV protection ability. The UPF value of the undyed silk fabric in this study was only 8.52. After dyeing with lac extract, the UPF of silk fabrics reached 28.56. The results showed that the UV resistance of the fabrics was improved after dyeing, and the UV resistance of the fabrics dyed with lac pigment was significantly improved. This is mainly owing due to the anthraquinone derivative molecular structure of the lac pigment, and the molecular structure contains more carbonyl, carboxyl, and hydroxyl groups, which can absorb high-energy ultraviolet light under the photochemical reaction, the molecular structure was reconstructed, and the ultraviolet light turned into other forms of energy, exhibiting a UV protection effect [34]. Mordant dyeing can promote the binding of lac pigment molecules and silk fabrics, increasing the content of the lac pigment molecules on silk fabrics to improve the UV protection performance.

#### 2.4.2. Antioxidant Activity

**Figure 6:**
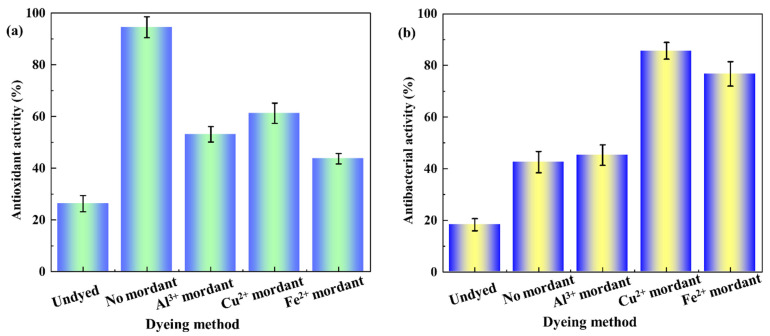
(**a**) Antioxidant and (**b**) antibacterial activity of dyed silk fabrics.

The silk fabric is in touch with the human body, so it is important for dyed silk fabric to have good oxidation resistance to enhance the health-related properties. Many natural dyes are phenolic hydroxyl compounds. In the antioxidation process of polyphenol compounds, phenolic hydroxyl groups react with free radicals to form stable semi-quinone free radicals, thus stopping the chain reaction and achieving antioxidation effects [12,13]. Figure 6a shows the antioxidant properties of the silk fabrics before and after dyeing. The antioxidant properties of the original silk were 26.35%, indicating that original silk shows poor antioxidant properties. The antioxidant property of the lac dyed silk fabrics is 98.57%, mainly because the lac pigment contains a large number of phenol hydroxyl groups, giving the dyed silk fabrics with good antioxidant function. After the three kinds of metal ion mordant dyeing, the antioxidation of the fabrics decreased significantly, indicating that the free radical scavenging ability of the silk fabrics was weakened. The main reason for this is that metal ions were complexed with the phenolic hydroxyl group in the process of mordant dyeing, resulting in the reduction in phenolic hydroxyl groups with a strong free radical scavenging ability, and thus reducing the antioxidant activity of dyed fabrics.

#### 2.4.3. Antibacterial Performances

Antibacterial activity is an important function of many natural dyes. Many reports give the research results that natural dyes can improve the antibacterial functions of textiles. Figure 6b shows the antibacterial properties of silk fabrics against *E. coli* before and after dyeing. Silk fabrics dyed with lac pigment have certain antibacterial properties (42.58%). The antibacterial properties here are mainly due to the lac pigment being a polyphenolic compound, and the phenolic hydroxyl group on the molecule has certain antibacterial properties, giving the silk fabrics a certain antibacterial effect [34]. After copper and ferric metal ion dyeing, the antibacterial effect of the dyed silk fabrics was significantly improved, reaching 85.68% and 76.72%, respectively. This is because the silk fabrics adsorb a large amount of copper and ferrous ions after mordant dyeing, which exhibits certain antibacterial property. This is especially the case for copper ions, which are known to have the excellent antibacterial properties found in metal ions [35].

The antibacterial and antioxidant properties of lac pigment and mordant dyed fabrics were compared with the fabrics dyed with other natural pigments, as presented in Table 4. As shown in the table, the reasonable antibacterial and antioxidant properties of lac-pigment-dyed fabrics indicated a potential application of lac pigment as the promising natural functional pigment.

## 3. Materials and Methods

### 3.1. Materials

Lac (origin: Xizang), silk fabrics (03 Crepe de Chine), purchased from Suzhou Jiadoli Silk Garment Co., Ltd. (Suzhou, Jiangsu, China). Anhydrous ethanol, glacial acetic acid, and sodium hydroxide were used. All reagents were procured from Sinopath Group Chemical Reagent Co., Ltd. (Shanghai, China). and were of analytical grade and required no further purification before use.

### 3.2. Extraction of Lac Pigment

The natural lac pigment was extracted from dried lac by immersion method. A certain amount of lac powder was put into a blue cap bottle, and various extraction tests were carried out in a constant temperature water bath. The effects of extraction solvent system (mixed solvent with different proportions of ethanol and water), solid–liquid ratio, pH value, temperature, time, and other single factors on the extraction efficiency were investigated, respectively, and the optimal extraction process conditions were obtained. In addition, under the same process parameters, lac pigment was repeatedly extracted until the color of the extraction liquid did not change significantly, and the extraction level and extraction rate were determined.

The extracted mixture is cooled and centrifuged for 10 min at 10,000 RPM using a high-speed centrifuge, and then filtered by vacuum to obtain the filtrate. The filtrate was diluted 50 times, and the spectrum in the range of 400–800 nm was obtained by UV-2450 UV–Vis spectrophotometer (Shimadzu (China) Co., Ltd., Shanghai, China). The absorbance at the maximum peak wavelength was observed to analyze the extraction solution.

### 3.3. Dyeing and Mordanting of Fabrics

Silk fabrics were dyed and functionalized with lac extraction by the infrared universal dyeing machine. The influence of dyeing parameters (temperature, concentration, and mordant) was studied by a single variable single time method to determine the best conditions for silk fabrics dyeing. Before dying, the silk fabric is treated in the standard environment [temperature (20 ± 2) °C, relative humidity 65% ± 2% for 24 h. All dyeing tests were performed with a bath ratio of 1:40. Start dyeing at 30 °C and heat up to a predetermined dyeing temperature with a heating rate of 2 °C/min, then dye at constant temperature for 60 min. After dyeing, rinse with tap water and dry at room temperature.

**Figure 7:**
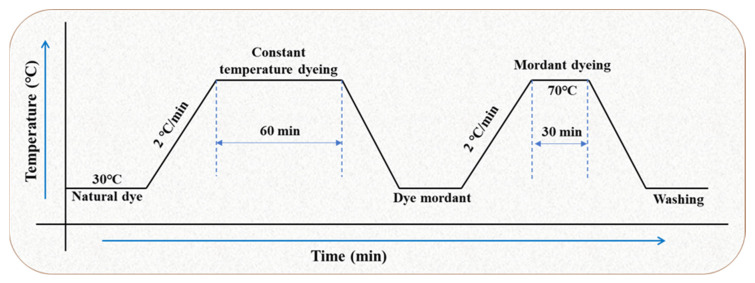
A process curve of step-by-step silk fabrics dyeing using natural dye.

The dyed fabric was treated with aluminum, copper, and ferrous metal salt solution, respectively. The mordant concentration is 2%. Dyeing was started at room temperature and the temperature was raised to 70 °C with a heating rate of 2 °C/min. Constant temperature mordant dyeing for 30 min. After the dyeing, the water was washed and dried. The overall process of dyeing experiment is shown in Figure 7.

### 3.4. Measurements and Characterizations

#### 3.4.1. Color Parameter Measurement

The color representation value L*a*b* value (brightness [L*], red/green light value [a*] and yellow/blue light value [b*]) and apparent color depth value (K/S value) of silk fabric were determined on the Datacolor 650TM (Guangzhou Chenglidong Instrument Co., Ltd., Guangzhou, China) computer color matching system with the following test conditions: D65 light source, 10° viewing angle. The color strength (K/S) was calculated using the following equation [3]:

$$K/S=\frac{1-R^{2}}{2R}$$

where K is the absorption coefficient, S is the scattering coefficient, and *R* is the reflectance.

#### 3.4.2. Durability Analysis of Dyed Fabrics

The color fastness to washing the dyed silk fabrics were carried out in the Wash Tec-P washing fastness machine in accordance with ISO 105-C06 [36]. Dyed silk fabrics are stitched together with two standard lining fabrics, placed in a mixture of soap and anhydrous sodium carbonate, mechanically agitated at a given time and temperature, and then washed and dried. Using dyed silk fabrics as a reference sample, the color change of the sample, and the stain of the lining fabric were evaluated by grey sample card.

The color fastness to rubbing was tested on the Model 670 color fastness to rubbing according to ISO 105-X12 [37]. Rub the white cloth back and forth over the dyed silk fabrics. After a certain number of frictions, the fastness to friction is analyzed by evaluating the degree of change in color compared to the white friction cloth.

The color fastness to light was carried out in accordance with ISO 105-B02 [38] on Atlas Xeno Test Alpha light fastness tester. The silk fabric is exposed under specified conditions under an artificial light source equivalent to daylight (D65), and then compared with the blue wool standard to assess its color fastness.

#### 3.4.3. Ultraviolet Protection Performance (UPF) Analysis

The UPF value and ultraviolet transmittance (T) of the dyed silk fabrics were determined by a UV-2000F Ultraviolet Transmittance Analyzer (Shanghai Ultra Blue Scientific Inc Technology Co. Ltd., Shanghai, China) in accordance with the AATCC 183 test procedure. Each sample was measured 5 times, and the average value was taken.

#### 3.4.4. Antioxidant Efficacy Assessment

The antioxidant activity was determined by the ABTS free radical achromatic assay reported in the literature. ABTS is dissolved in water and prepared into a solution of 7 mM concentration. ABTS radical positive ions (ABTS^+^) were obtained by the reaction of the prepared ABTS solution with the final concentration of 2.45 mM potassium persulfate solution, and then the mixed solution was placed in a dark room for 12–16 h. Free radicals remained stable at room temperature for more than two days in the dark room. Before use, the ABTS^+^ solution was diluted with phosphate buffer (0.1 m, pH 7.4) to the absorbance of 0.700 ± 0.025 at 734 nm, and 10 mg of dyed silk fabrics were added to the 10 mL ABTS^+^ solution. After 30 min, the absorbance was measured and the ability to scavenge free radical ABTS^+^ was calculated by Formula (1):

(1) $$\mathrm{Antioxidant}\;\mathrm{capacity}\;\left(\%\right)=\frac{A_{0}-A_{f}}{A_{0}}\times 100$$

where *A*_0_ is the absorbance of ABTS^+^ at the beginning, and *A*_f_ is the absorbance of ABTS^+^ after the fabric is immersed for 30 min.

#### 3.4.5. Antibacterial Activity

The antibacterial properties of the silk fabrics were evaluated following a modified AATCC 100-2004 test method [39]. *E. coli* (ATCC 43895) was selected for antibacterial performance assessment. The procedure was as follows: silk fabrics were pre-cut into 2.54 cm × 2.54 cm pieces and sterilized under UV light for 30 min. Place one of the sterilized fabrics in a Petri dish and add 25 μL of pre-cultured bacterial suspension at a specified concentration to the center of the fabric. Cover the fabric with another piece to ensure full contact between the two film layers and the bacteria. After 60 min of contact, remove the fabric and transfer it to a sterile centrifuge tube containing 5 mL of PBS solution. Vortex the mixture for 2 min. Take 0.1 mL of the vortexed solution, perform gradient dilutions using PBS solution, and inoculate the diluted bacterial solution onto agar culture medium. Incubate the agar plates at 37 °C in a constant temperature incubator for 24 h. After incubation, assess the antibacterial effectiveness by counting colony-forming units (CFUs). The reduction in bacterial growth of the tested fabrics was calculated using the following equation:

(2) $$\mathrm{Antibacterial}\;\mathrm{capacity}\;\left(\%\right)=\frac{N_{c}-N_{f}}{N_{c}}\times 100$$

where *N*_c_ is the number of colonies formed on the Petri plate of pre-cultured bacterial suspension and *N*_f_ is the number of colonies formed on the Petri plate of fabric.

## 4. Conclusions

In this work, owing to the study of Tibetan traditional national dyeing and weaving culture, lac extract was found to be a potential functional natural dye. The extraction technology of lac pigment was optimized by studying the influence of extraction parameters on the extraction rate. The results showed that the optimal extraction technology of lac pigment was as follows: the volume ratio of ethanol to water was 60:40 with the solid–liquid ratio of 1:10, and the extraction was reacted at 70 °C for 1 h under the neutrality condition. In addition, lac pigment exhibits a certain lifting force on the dyeing of silk fabrics, which is suitable for darkness. The antioxidation and anti-ultraviolet properties of the dyed silk fabrics were greatly improved, the antioxidation property was 98.57%, and the UPF value was 28.56. Different post-mordant experiments showed that the color of the fabrics was brighter after dyeing with Al^3+^ mordant, and it displayed excellent fastness in terms of washing, friction, sun, and UV (UPF value was 42.68). The silk fabrics dyed with lac pigments proved to be promising, and may eventually become a viable option that can meet the current needs of the textile industry for sustainable silk dyeing and functionalization.

## Figures and Tables

**Table 1:** Effect of metal ion coordination on the K/S value and color characteristic value of lac red dyed silk fabrics.

Dyeing Method	λmax	K/S Value	L	a	b
No mordant	440	3.03	49.44	19.65	9.55
Al^3+^ mordant	530	4.80	42.82	23.11	−0.8
Cu^2+^ mordant	430	3.22	46.50	9.89	3.06
Fe^2+^ mordant	430	4.90	40.76	3.45	4.01

**Table 2:** Color fastness of lac extract dyed silk fabrics before and after mordant dyeing.

Dyeing Method	Rubbing Fastness		Washing Fastness		Light Fastness
	Dry	Wet	Fade	Staining	
No mordant	3	2–3	2	3	4
Al^3+^ mordant	4–5	4	4	4–5	4–5
Cu^2+^ mordant	4–5	4	3–4	4	4–5
Fe^2+^ mordant	4	4	3–4	4–5	4

**Table 3:** Effects of different mordants on the UV resistance of silk fabrics.

Dyeing Method	UPF	T (UVA, %)	T (UVB, %)
Undyed	8.52	11.14	5.21
No mordant	28.56	4.03	4.57
Al^3+^ mordant	42.68	1.97	2.01
Cu^2+^ mordant	35.61	2.94	2.85
Fe^2+^ mordant	38.91	2.03	2.08

**Table 4:** Antibacterial and antioxidant properties of fabrics dyed with natural dyes.

Dye	Antibacterial		Antioxidant	References
	*S. aureus*	*E. coli*		
Gardenia yellow	100%	100%	38%	(Wang et al., 2024) [2]
Blue bio-colorant phycocyanin	/	/	70%	(Wu et al., 2023) [14]
Gardenia yellow	>95%	>95%	/	(Wang et al., 2023) [6]
Nigerian mango leaves	/	/	80%	(Jabar et al., 2023) [24]
Lac	40.8%	69.11%		(Do et al., 2023) [34]
Flavonoid dyes from vine tea	64.7%	73%	45.13%	(Zhang et al., 2022) [11]
Black rice extract	>80%	>80%		(Haque et al., 2022) [4]
Lac	/	42.58%	98.57%	This work
Lac + Cu^2+^	/	85.68%	61.27%	This work

## Data Availability

Data are contained within the article.
